# NEAP/DUSP26 suppresses receptor tyrosine kinases and regulates neuronal development in zebrafish

**DOI:** 10.1038/s41598-017-05584-7

**Published:** 2017-07-12

**Authors:** Chi-Hwa Yang, Yu-Jung Yeh, Jiz-Yuh Wang, Ya-Wen Liu, Yen-Lin Chen, Hui-Wen Cheng, Chun-Mei Cheng, Yung-Jen Chuang, Chiou-Hwa Yuh, Yi-Rong Chen

**Affiliations:** 1 Institute of Molecular and Genomic Medicine, National Health Research Institutes, Zhunan, 350 Taiwan; 2 Institute of Bioinformatics and Structural Biology, National Tsing Hua University, Hsinchu, 300 Taiwan; 3 Graduate Institute of Medicine, College of Medicine, Kaohsiung Medical University, Kaohsiung, 807 Taiwan

## Abstract

Expression of neuroendocrine-associated phosphatase (NEAP, also named as dual specificity phosphatase 26, [DUSP26]) is restricted to neuroendocrine tissues. We found that NEAP, but not its phosphatase-defective mutant, suppressed nerve growth factor (NGF) receptor TrkA and fibroblast growth factor receptor 1 (FGFR1) activation in PC12 cells upon NGF stimulation. Conversely, suppressing NEAP expression by RNA interference enhanced TrkA and FGFR1 phosphorylation. NEAP was capable of de-phosphorylating TrkA and FGFR1 directly *in vitro*. NEAP-orthologous gene existed in zebrafish. Morpholino (MO) suppression of NEAP in zebrafish resulted in hyper-phosphorylation of TrkA and FGFR1 as well as abnormal body postures and small eyes. Differentiation of retina in zebrafishes with NEAP MO treatment was severely defective, so were cranial motor neurons. Taken together, our data indicated that NEAP/DUSP26 have a critical role in regulating TrkA and FGFR1 signaling as well as proper development of retina and neuronal system in zebrafish.

## Introduction

Proteins of the nerve growth factor (NGF) family, also known as neurotrophins (NTs), are evolutionary conserved factors with numerous functions. NTs (including NGF, brain-derived growth factor [BDGF], NT3, and NT4/5) stimulate signals through receptor tyrosine kinases (RTKs, including TrkA, B, and C) and/or p75NTR, a tumor necrosis factor receptor family member. These two distinct classes of receptors are preferentially activated by mature NT and unprocessed pro-NT, respectively^1–3^. Due to the earlier identification, more information is known about the NGF-induced signaling and its biological effects mediated by TrkA and p75NTR^1^. Sympathetic neurons express both p75^NTR^ and TrkA; while pro-NGF activates p75NTR and induces cell death, mature NGF activates both p75NTR and TrkA to promote cell survival^1, 3^. Signaling events triggered by Trk receptors are similar to those of other RTKs; however, Trk-containing endosomes delivering the activated signaling complex to the distant somas through retrograde axonal transportation is a unique feature in nerve cells^2, 4^.

Phosphorylation is a prominent post-translational modification in proteins and its levels are balanced by actions of kinases and phosphatases. Dual-specificity phosphatases (DUSPs) are particularly interesting among all protein phosphatases^5–7^. DUSPs, defined by sequence homology to MAP kinase phosphatases (MKPs), are named for their ability to de-phosphorylate both serine/threonine and tyrosine residues. Some DUSP members have been classified as atypical DUSPs due to the presence of a conserved DUSP phosphatase domain while lacking other recognizable domains and showing little or no activity against major MAPKs^5–7^.

We previously identified an atypical DUSP named neuroendocrine-associated phosphatase (NEAP), also named as DUSP26 or MKP-8^8–10^. We found that NEAP/DUSP26 is specifically expressed in neuroendocrine cells/tissues and does not inactivate MAPKs^9^. NEAP/DUSP26 suppresses NGF-induced PC12 differentiation through down-regulating the phosphatidylinositide-3 kinase (PI3K)/Akt pathway and impairs PC12 cell growth through suppressing EGFR expressions by WT1-mediated transcriptional suppression^9, 11^. Nevertheless, other reports find that NEAP/DUSP26 is capable of inactivating p38-MAPK^8, 12^. NEAP/DUSP26 dephosphorylates p53 and inhibits its tumor suppressor activity in neuroblastoma cells^13^. Adenylate kinase 2 (AK2) has been shown to enhance NEAP/DUSP26 activity against FADD and suppress cell growth^14^. A recent report shows that NEAP/DUSP26 promotes Aβ42 production through enhancing axonal transportation of amyloid precursor protein during hypoxia^15^.

In this study we found that NEAP/DUSP26 expression decreased TrkA and FGFR1 phosphorylation induced by NGF and, conversely, knockdown of NEAP enhanced TrkA and FGFR1 activation. NEAP/DUSP26 dephosphorylated TrkA and FGFR1 directly. Suppression of NEAP/DUSP26 expression in zebrafish embryos also caused activation of TrkA and FGFR1 as well as defects in retinal and neuronal development. Our data supported a critical role of NEAP/DUSP26 in regulating RTK signaling in neuronal system.

## Results

### NEAP decreases NGF-, but not heregulin-, induced Akt activation in PC12 cells

**Figure 1:**
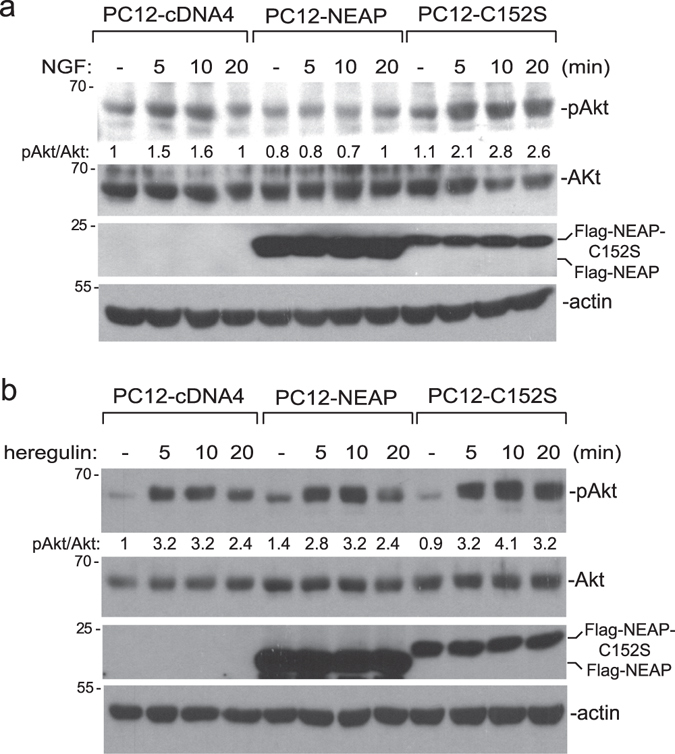
NEAP suppresses NGF-, but not heregulin-, induced Akt phosphorylation. (**a** and **b**) PC12-cDNA4, NEAP, or NEAP-C152S cells were treated with NGF (50 ng/ml) and the cells were collected at the indicated time points. Proteins of interest in the extracts were examined by immune-blotting assays using specific antibodies. Phospho-protein intensity was normalized by the level of the corresponding protein in the same sample. The phospho-protein/protein ratio in the untreated PC12-cDNA4 cells was designed as 1. The results from two independent experiments were presented in Supplementary Materials.

We previously showed that NEAP down-regulated NGF-induced Akt activation specifically without affecting other signaling pathways, such as ERK, JNK, and p38-MAPK. However, further examinations showed that PI3K/Akt pathway was not directly targeted by NEAP^9^. To know whether NEAP could down-regulate the PI3K/Akt pathway in response to different stimuli, we treated PC12-cDNA, NEAP, and NEAP-C152S (phosphatase-inactive mutant) cells with either NGF or heregulin and examined for Akt phosphorylation. We found that although NEAP suppressed NGF-induced Akt phosphorylation at Ser 473, it did not decrease heregulin-induced Akt phosphorylation as much in the same PC12 cell system (Fig. 1). This result indicated that, instead of targeting PI3K/Akt, NEAP must have acted on a molecule(s) more upstream in the TrkA signaling pathway.

### NEAP suppresses TrkA and FGFR1 phosphorylation in PC12 cells upon NGF stimulation

**Figure 2:**
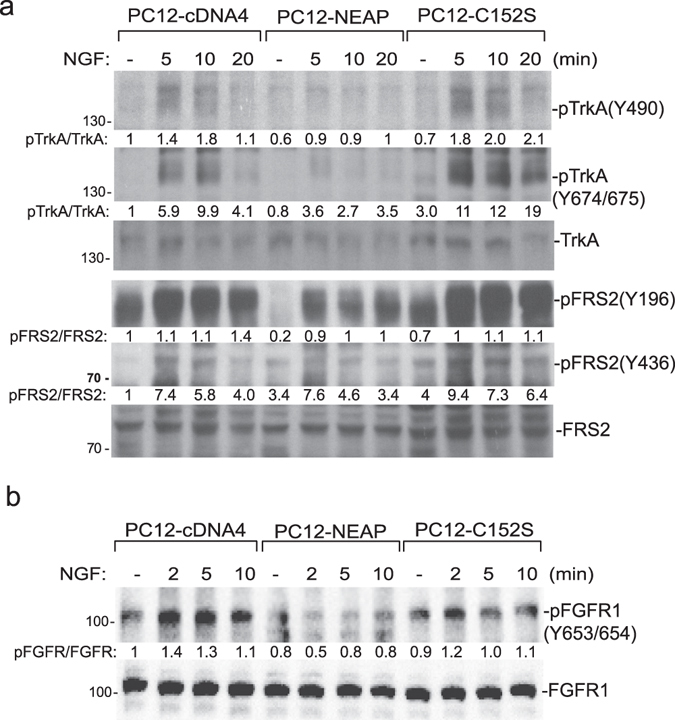
NEAP suppresses NGF-induced TrkA and FGFR1 phosphorylation. PC12-cDNA4, NEAP, or NEAP-C152S cells were treated with NGF (50 ng/ml) and the cells were collected at the indicated time points. (**a**) Levels of TrkA, pTrkA, FRS2, and pFRS2 were examined by immune-blotting assays using specific antibodies as indicated. (**b**) Levels of FGFR1 and pFGFR1 were examined by immune-blotting assays. The phospho-protein/protein ratio in the untreated PC12-cDNA4 cells was designed as 1. The results from two independent experiments were presented in Supplementary Materials.

The results shown in Fig. 1 prompted us to examine whether NGF receptor TrkA was directly targeted by NEAP. We found that TrkA phosphorylations on Tyrs 490 and 674/675 were decreased by NEAP, but not by the phosphatase-inactive mutant (NEAP-C152S), upon NGF stimulation. Phosphorylation of FRS2 (at Tyrs 196 and 436), an adaptor molecule mediating TrkA signaling^16^, was also decreased in the presence of NEAP (Fig. 2a). FGFR1 is known to be activated by NGF signaling through receptor cross talk and autocrine effect^17^. We found that NGF-induced FGFR1 phosphorylation on Tyr 653/654 was also decreased by NEAP expression in PC12 cells (Fig. 2b).

**Figure 3:**
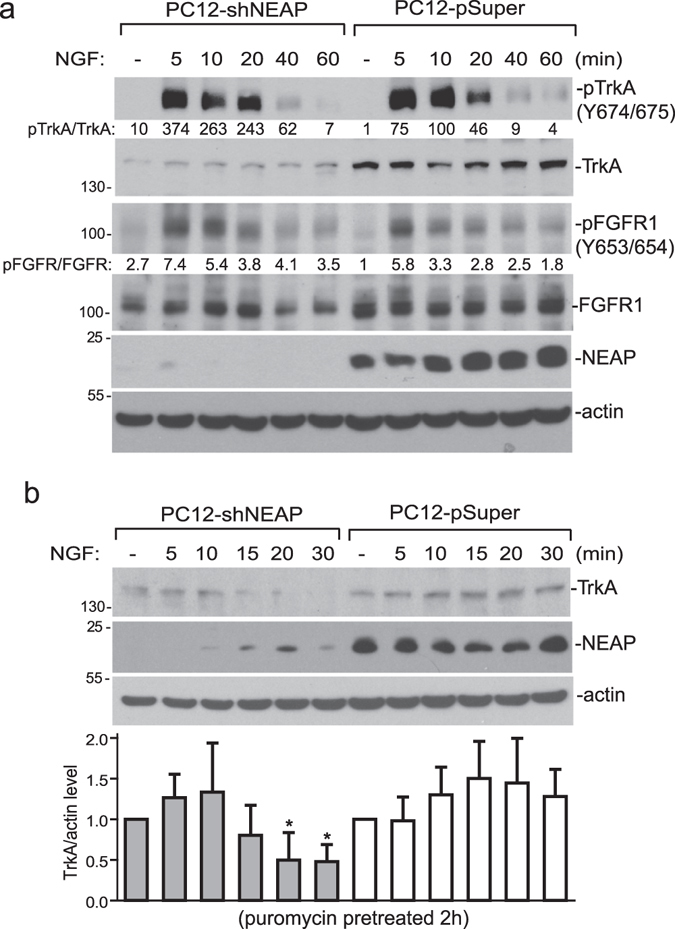
Suppression of NEAP expression increases NGF-induced TrkA and FGFR1 phosphorylation. PC12-pSuper and PC12-shNEAP cells were treated with NGF (50 ng/ml) and the cells were collected at the indicated time points. (**a**) Levels of TrkA, pTrkA, FGFR1 and pFGFR1, and NEAP were examined by immune-blotting assays using specific antibodies as indicated. The pTrkA/TrkA and pFGFR1/FGFR1 ratios in the untreated PC12-pSuper cells were designed as 1. The results from two independent experiments were presented in Supplementary Materials. (**b**) The cells were pre-treated with puromycin (5 μg/ml) for 2 h and then stimulated with or without NGF (50 ng/ml) for the indicated time periods. The levels of TrkA, NEAP, and β-actin were examined by immune-blotting assays. The ratios of TrkA/actin were determined for each sample and its values in untreated PC12-pSuper and shNEAP cells were designed as 1. The results (mean ± standard deviation) from three immuno blots were analyzed by the Student’s t test. The time points with significantly lower TrkA expression (*p* < 0.05) in comparison with the untreated control were indicated with asterisks.

Suppressing NEAP expression by the RNA interference method decreased the levels of both TrkA and FGFR1 (Fig. 3a). In this reversed experiment, both NGF-induced TrkA and FGFR1 Tyr phosphorylation (normalized by receptor levels) were elevated in PC12-shNEAP cells in comparison to those in PC12-pSuper control cells (Fig. 3a). TrkA is down-regulated by ubiquitination and degradation upon ligand stimulation and phosphorylation^18, 19^. In light of the low level of TrkA in PC12-shNEAP cells, we examined whether lack of NEAP caused a quicker turnover of TrkA protein. Upon inhibition of protein synthesis by puromycin, NGF-induced TrkA degradation indeed was faster in PC12-shNEAP cells in comparison to that in PC12-pSuper control cells (Fig. 3b). Our data clearly indicated that TrkA, or its closely associated signaling molecule(s), was targeted by NEAP in the PC12 cell system.

### NEAP de-phosphorylates TrkA and FGFR1 directly *in vitro*

**Figure 4:**
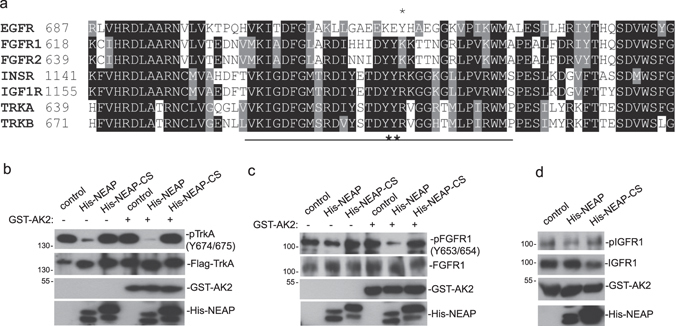
NEAP dephosphorylates TrkA and FGFR1 directly. (**a**) Activating loop sequences of EGFR, FGFR1, FGFR2, IGFR1, INSR, TrkA, and TrkB were aligned using the Clustal Omega and Boxshade programs. Identical and homologous amino acid residues were shown by black and grey shades, respectively. Dual Tyr-phosphorylation sites were indicated by bold asterisks. EGFR Tyr 845 was indicated by an asterisk. (**b**) Flag-tagged pTrkA was immune-precipitated from transfected H1299 cells and was subjected to NEAP dephosphorylation with or without the GST-AK2 presence. The levels of pTrkA/TrkA in the reaction mixtures were examined by immune-blotting using indicated antibodies. (**c** and **d**) Rat FGFR1 and IGFR1 were immunoprecipitated from PC12 cells and subjected to NEAP dephosphorylation in the presence or absence of GST-AK2. The levels of pFGFR1/FGFR1 (panel c) and pIGFR1/IGFR1 (panel d) in the reaction mixtures were examined by immune-blot analyses using indicated antibodies.

Tyr residues 674 and 675 are located at the activating loop of the TrkA kinase domain and are important for NGF-induced biological functions^20^. Phosphorylation(s) at the activating loop is a common feature for kinase activation and the similar dual Tyr phosphorylation were found in the activating loops of FGFR and IGFR family members, but not in EGFR (Fig. 4a)^21, 22^. In light of the data in the PC12 cell system, we tested whether TrkA, FGFR1, and IGFR1 were direct substrates of NEAP *in vitro*. As shown in Fig. 4b, NEAP, but not NEAP-C152S mutant, dephosphorylated TrkA on Tyr 674/675 *in vitro*. The addition of GST-AK2, a previously reported NEAP-associated protein^14^, enhanced the de-phosphorylation ability (Fig. 4b). Similarly, NEAP could de-phosphorylate FGFR1 either in the presence or absence of AK2 (Fig. 4c). However, only a minimal de-phosphorylation effect on IGFR1 was observed under the same condition (Fig. 4d). The data of *in vitro* reactions showed that TrkA and FGFR1 were directly de-phosphorylated by NEAP.

### NEAP is expressed in the central nerve system of zebrafishes

**Figure 5:**
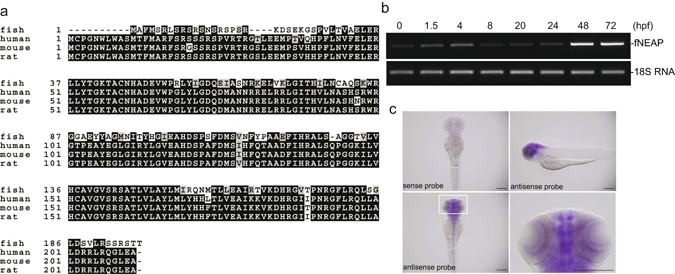
Zebrafish NEAP is expressed in central nerve system. (**a**) Alignment of zebrafish (fish), human, mouse, and rat NEAP protein sequences was performed using the Clustal Omega and BoxShade programs. (**b**) Expression levels of NEAP and 18S ribosomal RNA (18S RNA) in zebrafish embryos collected at the indicated time points were determined using the RT-PCR method. (**c**) Expression pattern of zebrafish NEAP (shown in purple color) was determined using the *in situ* hybridization assay as described in the Methods. The inset of the lower left panel was enlarged in the lower right panel to show the positive hybridization signals in the eyes. Scale bar: 0.2 mm.

We then used zebrafish as an experimental system to study the biological role of NEAP *in vivo*, since NEAP orthologous gene can be found in zebrafish and its protein was highly conserved (with sequence 73% homologous and 64% identical to human NEAP) in comparison to those of rodents and human (Fig. 5a). NEAP mRNA was detectable in the zebrafish eggs at 0 h post fertilization (hpf). The expression levels of NEAP increased evidently at 48 hpf, which is later than the development of neuromeres (at 18 hpf) and the distinctive morphogenesis of central nerve system (CNS) at 24 hpf^23^, in the zebrafish embryos (Fig. 5b). Using the whole mount *in situ* hybridization (WMISH) assay, we detected NEAP mRNA expression most strongly in the brain of zebrafish embryos (Fig. 5c). NEAP mRNA could also be detected in zebrafish retina (Fig. 5c, lower right panel). These results were consistent with our previous report showing that NEAP is preferentially expressed in human neuroendocrine tissues^9^.

### Suppression of NEAP leads to hyper-phosphorylation of TrkA and FGFR1

**Figure 6:**
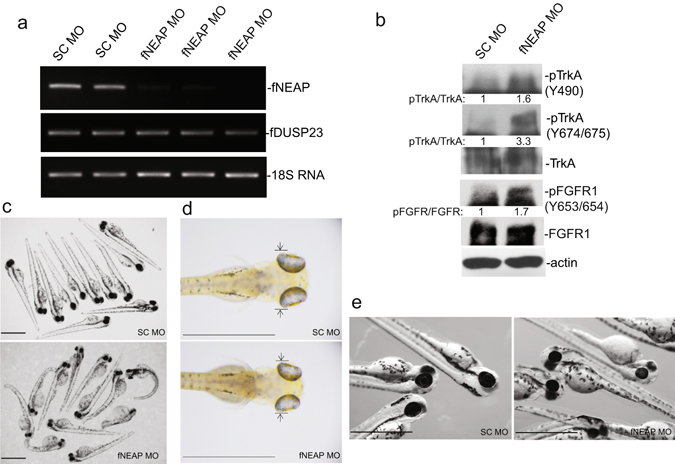
Suppression of NEAP expression in zebrafish causes biochemical and morphological abnormalities. (**a**) Zebrafish fertilized eggs were injected with a morpholino (MO) against fNEAP or a corresponding control MO with scrambled sequence (SC). The expression of fNEAP, fDUSP23, and 18S RNA at the 72-hpf time point were examined by RT-PCR. (**b**) Fish embryos treated with SC or fNEAP MO were subjected to extract preparation and immune-blotting analyses using the indicated antibodies. (**c**) General abnormality of zebrafishes with MO treatments at the 72-hpf time point. (**d**) Head widths of MO-injected zebrafish were measured as indicated by the arrows. (**e**) Smaller eyes and heads in fNEAP MO-treated zebrafishes. Twelve MO-injected fishes from each group were randomly selected for measuring the head and eye sizes. Scale bars: 1 mm.

We then suppressed NEAP expression in zebrafish embryos using the morpholino (MO) approach. As shown in Fig. 6a, a MO targeting the fish NEAP mRNA decreased the expression of NEAP but not that of a homologous DUSP23. Knockdown of NEAP was associated with increased phosphorylation of TrkA and FGFR1 in zebrafish embryos, suggesting that these two receptor tyrosine kinases (RTKs) were regulated by NEAP *in vivo* (Fig. 6b). In general, NEAP MO injection had various effects on embryos. Some but not all of the injected fishes showed abnormal body curvature (Fig. 6c). However, all of the NEAP MO morphants had significantly smaller head (468 ± 48 vs. 550 ± 9 μm, p < 0.001; Fig. 6d) and smaller eyes (172 ± 44 vs. 260 ± 27 μm, p < 0.001; Fig. 6e) in comparison to those of fish embryos treated with control scrambled (SC) MO.

### Zebrafishes lacking NEAP have developmental defects in retina and neuronal system

**Figure 7:**
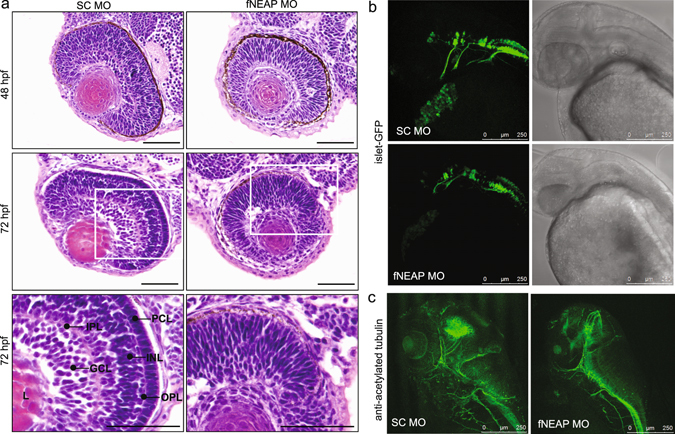
NEAP deficiency causes defects in retina and motor neuron development. (**a**) Zebrafish embryos treated with SC or fNEAP MO (12 ng) were collected at 48 or 72 hpf and subjected to histological analyses as described in the Methods. The development of zebrafish retina was shown with hematoxilin & eosin staining. Scale bars: 50 μm. Abbreviations: gcl, ganglion cell layer; inl, inner nuclear layer; ipl, inner plexiform layer; opl, outer plexiform layer; pcl, photoreceptor cell layer. (**b**) Islet-GFP transgenic zebrafish embryos treated with SC or fNEAP MO (12 ng) were subjected to microscopic analyses. The development of fluorescent cranial motor neurons and head morphology were documented by fluorescent (left panels) and phase contrast (right), respectively. (**c**) Zebrafish embryos treated with SC or fNEAP MO were subjected to *in situ* immune-fluorescence staining using an anti-acetyl tubulin antibody.

The smaller eyes and heads in NEAP MO morphants were intriguing. Hematoxylin & eosin staining and microscopic examinations showed that the NEAP MO caused defective retinal development. Unlike the zebrafishes with SC MO had differentiated and layered retina, NEAP MO morphants’ retina containing cells without distinctive layers even at 72 hpf (Fig. 7a). Also, the eye lens of NEAP morpholino-treated fishes were less developed than that of the control group (Fig. 7a). This result indicated that NEAP had important functions in regulating retina and eye development.

Additionally, smaller heads suggested the development of central nerve system (CNS) was defective in zebrafishes lacking NEAP. We first examined the development of cranial motor neurons using the islet-GFP transgenic fishes, because their GFP-labeled neurons can be visualized through emitting green fluorescence^24^. In NEAP MO/islet-GFP morphants, which has smaller heads, cranial motor neurons (showed in green) did not show long axonal extensions in comparison to those in control group (Fig. 7b). Consistent with this, whole mount *in situ* fluorescence staining using an anti-acetylated tubulin antibody showed a similar result that there was less axonal structures in NEAP MO-injected zebrafishes (Fig. 7c). These collective data indicated that NEAP was essential for normal eye and CNS development in zebrafishes.

## Discussion

Previously we found that NEAP/DUSP26 suppressed NGF-induced PI3K/Akt activation in PC12 cells without affecting MAP kinase pathways. When ectopically expressed in H1299 cells, NEAP/DUSP26 also failed to inhibit the activation of all MAPKs^9^. However, we also do not find any evidence that NEAP acts on PI3K or Akt directly^9^. Moreover, NEAP/DUSP26 failed to suppresses heregulin-induced Akt activation in PC12 cells, suggesting that, instead of being an Akt suppressor, NEAP/DUSP26 must act on TrkA or TrkA-associated signaling molecules. Using antibodies generated against phosphorylated motifs of TrkA, we found that Tyr-phosphorylation levels of TrkA were decreased in PC12 expressing NEAP/DUSP26, but not its phosphatase-defective mutant. Phosphorylation of FRS2, an adaptor molecule required for proper TrkA signaling^16^, was also decreased in the presence of NEAP/DUSP26. It is a bewildering question that why expression of NEAP specifically affected PI3K/Akt pathway but not other TrkA-downstream signaling pathways and we do not have a definite answer at this point. One possible explanation is that all MAPK pathways are consisted of multiple layers of kinases and the initial suppressive effect of NEAP/DUSP26 is diminished by the amplification nature of kinase cascades.

Other than TrkA, we found NGF-induced FGFR1 phosphorylation was also suppressed by NEAP/DUSP26. FGFR1 activation through autocrine production is critical for proper neurite outgrowth in PC12 cells upon NGF treatment^17^. However, the immediate response of FGFR1 phosphorylation to NGF stimulation implicated that cross talk between TrkA and FGFR1 existed in the PC12 context (Fig. 2). We noticed that the dual Tyr-phosphorylation motifs in the activating loops are conserved among the neurotrophin receptor and FGFR families. It is possible that sequence homology among these receptors allowed selective substrate recognition by NEAP/DUSP26. Nevertheless, despite the result that NEAP/DUSP26 directly de-phosphorylated TrkA and FGFR1 *in vitro*, it did not de-phosphorylate IGFR1 whose activating loop also has the conserved dual Tyr-phosphorylation motif. The mechanism determines that substrate selectivity of NEAP/DUSP26 is very intriguing. It has been shown that adenylate kinase 2 (AK2) interacts with NEAP/DUSP26 and facilitates the de-phosphorylation of FADD^14^. We have tested the combination of AK2 and NEAP/DUSP26 *in vitro* to see whether AK2 has a role in mediating NEAP/DUSP26’s effect on RTKs and did find an enhancing NEAP/DUSP26 activity against pTrkA in the presence of AK2. However, we did not detect the NEAP-AK2 interaction in PC12 cells by various methods. Therefore, what determines the substrate selectivity of NEAP/DUSP26 against RTKs in neuronal cells remained unclear. Moreover, despite detecting a direct de-phosphorylation of FGFR1 by NEAP *in vitro*, we cannot conclude whether NEAP decreased NGF-induced FGFR1 phosphorylation through direct, indirect, or the combination of both mechanisms at this point.

The NEAP/DUSP26-orthologous gene was found in zebrafish and was highly expressed in the central nerve system. The noticeable curved body posture and smaller eyes and heads in NEAP/DUSP26 morpholino-injected embryos indicated that this gene was important in the developmental process. Defective differentiation of retina and failure in axonal extension of cranial motor neurons further supported the role of NEAP/DUSP26 in the neuronal system. Suppression of NEAP/DUSP26 by morpholino injection up-regulated the Tyr phosphorylation levels of TrkA and FGFR1 in zebrafish embryos, which was consistent with our finding *in vitro*. However, we cannot conclude that the developmental defects were exclusively caused by aberrant RTK signaling in light of the fact that other known NEAP/DUSP26’s substrates also have important roles in neuronal cell differentiation and survival^12–14, 25^. Moreover, TrkA is known to be a substrate of other protein Tyr phosphatases (PTPs)^26, 27^. It is also possible that other PTPs can compensate, in parts, the loss of NEAP/DUSP26 *in vivo*. NEAP/DUSP26 expression was not paralleled with the initial development of nerve system and had the highest expression at a later developmental stage in zebrafish embryos. Interestingly, NEAP/DUSP26 expression is also highest in differentiated PC12 cells with well formed neurites, indicating a role of NEAP in mature neuronal cells. More detailed analyses of genetically modified animal models will facilitate the understanding of the biochemical and biological function of NEAP/DUSP26.

## Methods

### Cell culture and transfection

Establishment of permanent PC12 cell lines, including PC12-cDNA4, PC12-NEAP, PC12-C152S, PC12-pSuper and PC12-shNEAP (previously named PC12-NEAPi) were described previously^9^. Parental PC12 and its derivative cell lines were maintained as monolayer cultures in a 100-mm culture dish in Dulbecco’s modified Eagle’s medium (DMEM) supplemented with 10% horse serum (HS), 5% heat inactivated fetal bovine serum (FBS), penicillin (50 unit/ml) and streptomycin (50 μg/ml). H1299-derivative cells were cultured in RPMI medium supplemented with 10% FCS plus penicillin and streptomycin and were transfected using the Lipofectamine 2000 reagent.

### Plasmid constructions

Rat TrkA-coding DNA fragment was PCR amplified using specific primers and a PC12 cDNA pool as a template. The TrkA fragment was then inserted between BamHI and EcoRI sites in pcDNA4/TO/myc-His B vector (Invitrogen, Carlsbad, CA). Recombinant His-tagged NEAP was constructed by inserting the NEAP-coding sequence between the Bam HI and Xho I sites of pHis-4T-3 vector. AK2 DNA fragment was PCR-amplified using specific primers and a H1299 cDNA pool as a template. Recombinant GST-AK2 expressing vector was constructed by inserting the AK2-coding sequence between the Bam HI and Xho I sites of pGST-4T-3 vector. Zebrafish NEAP coding sequence (reference Genbank number XM_694337) was PCR-amplified, using a zebrafish cDNA pool as a template, and was then inserted between BamHI and Xho I sites of the pCMV-Tag2B vector, which contains T3 and T7 promoters for the synthesis of sense and antisense probes, respectively.

### Reagents and antibodies

Anti-Flag (M2), anti β-actin, and anti-acetylated tubulin monoclonal antibodies were purchased from Sigma (St. Louis, MO, USA). Anti-NEAP/DUSP26 antibody was purchased from GeneTex (Hsinchu, Taiwan). Anti-phospho-Akt (S473), pTrkA (Y490 and Y674/675), anti-pFGFR1(Y653/654), anti-pFRS2 (Y196 and Y436), anti-pIGFR1(Y), and anti-IGFR1 antibodies were purchased from Cell Signaling Technology (Beverly, MA, USA). Anti-Akt, anti-TrkA, anti-FGFR1, anti-GST antibodies and peroxidase-conjugated anti-mouse and anti-rabbit IgG antibodies were purchased from Santa Cruz Biotechnology (Santa Cruz, CA, USA). NGF was purchased from R & D System (Minneapolis, MN, USA) and heregulin was purchased from Calbiochem (San Diego, CA, USA).

### Cell extract preparation and immunoblotting analysis

Whole cell lysate was prepared by suspending 2 × 10^6^ cells in 200 μl of lysis buffer (50 mM Tris [pH 8.0], 150 mM NaCl, 1% triton-X 100, 0.5% deoxycholate, 0.1% SDS, 2 μg/ml leupeptin, 5 μg/ml aprotinin, 1 mM PMSF, 1 mM DTT, and 1 mM Na_3_VO_4_). Immunoblotting assays were performed as described previously^9^.

### Immuno-precipitation (IP) and phosphatase assays

Target proteins in cell extracts were precipitated by incubation with a specific antibody plus protein A/G-agarose beads in 1 ml of lysis buffer with continuously rotation at 4 °C for 2 h. The precipitates were washed three times with lysis buffer. IP complexes for phosphatase assays were washed twice more with phosphatase reaction buffer (50 mM Tris (pH 7.0), 50 mM Bis-Tris, 100 mM sodium acetate, and 10 mM DTT). The immune-complex was then mixed with 100 μl of phosphatase reaction buffer containing 20 μg/ml of GST-NEAP or GST-NEAP (C152S), in the presence or absence of GST-AK2 (20 μg/ml). The phosphatase reaction was performed at 37 °C for 1 h, and then terminated by adding SDS sampling buffer. The reaction mixtures were heated at 95 °C for 5 min and analyzed by SDS-PAGE plus immunoblotting blot analyses.

### Zebrafish morpholino (MO) injection and embryo examination

Zebrafish NEAP MO and scrambled MO were purchased from Gene Tools, LLC (Philomath, OR), dissolved in sterilized ddH_2_O (17.39 µg/µl), and stored at −20 °C. For microinjection, MOs were prepared in phosphate buffered saline with 0.05% phenol red and then were injected into embryos of one-cell stage with PV820 Pneumatic PicoPump (World Precision Instruments, Inc., Sarasota, FL). After injection, embryos were incubated in egg water (60 µg sea salt/ml distilled water) supplemented with 0.003% 1-phenyl-2-thiourea (PTU) at 28 °C to prevent pigmentation. Embryo development was examined at 1.5, 4, 8, 20, 24, 48 and 72 hpf.

### Whole mount *in situ* hybridization (WMISH) and immune-fluorescence (IF) staining

Embryos were fixed in 4% paraformaldehyde overnight at 4 °C and dehydrated in methanol at −20 °C. WMISH procedure was performed according to the protocol described by Thisse B and Thisse C on ZFIN (http://zfin.org) with some modifications. In brief, embryos were rehydrated gradually with PBST (1x PBS, 0.1% Tween 20). For 96-hpf embryos, they were digested with 25 µg/ml proteinase K (Sigma-Aldrich) in PBST for 30 minutes at room temperature. After post-fixed with 4% paraformaldehyde, the embryos were incubated in HYB^+^ (50 µg/ml heparin, 500 µg/ml wheat germ tRNA and HYB^−^ containing 50% formamide, 5x SSC and 0.1% Tween 20) which contained 100 ng DIG-labeled antisense (or sense) RNA probe at 65 °C overnight. Embryos were then washed with 75%, 50% and 25% HYB^−^/2x SSC for 10 minutes, 2x SSC for 10 minutes, two times with 0.2x SSC for 30 minutes, 75% 0.2x SSC/25% PBST, 50% 0.2x SSC/50% PBST, 25% 0.2x SSC/75% PBST for 5 minutes at 65 °C and PBST for 10 minutes and transferred to blocking buffer (2 mg/ml BSA, 2% sheep serum in PBST) at least for 1 hour. Embryos were incubated in 1∶5000 anti-DIG-AP (Roche) in blocking buffer overnight at 4 °C. After 6 washes with PBST for 15 minutes and 3 washes with alkaline Tris buffer for 5 minutes, bound antibody was detected by SIGMA *FAST*™ BCIP/NBT. After staining, labeled embryos were mounted in 90% glycerol and examined on an Olympus stereomicroscope (SZX-ILLD100, Tokyo, Japan). The images were captured using an Olympus DP70 digital microscope camera and processed by Helicon Focus software. Whole mount IF staining using the anti-acetylated tubulin antibody was performed as described previously^28^.

### RNA extraction and RT-PCR

The RNA extraction was performed using the Trizol reagent (Thermo Fisher Scientific) in combination with the MagNA Lyser Green Beads (Roche Diagnostics, Indianapolis, IN) according to the instructions of the manufacturer. Reverse transcription of cDNAs were performed using the ProtoScript first strand cDNA synthesis kit (New England Biolabs, Beverly, MA) and using 1 μg of total RNA as templates. PCR reactions were performed and the reaction products were analyzed using agarose gel electrophoresis.

### Nucleotide sequences

The nucleotide sequences used in this study are listed below.

Rat TrkA forward: 5′-GCGGAATTCGCCACCATGCTGCGAGGCCAGCGGCAC,

TrkA reverse: 5′-GCGCTCGAGATGCCCAGAACGTCCAGGTAACTC,

NEAP forward: 5′-GCGGGATCCACCATGGCCCGCTTCTCCCGGAG,

NEAP reverse: 5′-GCGGTCGACATTGCTTCCAGACCCTGCCGCAG,

AK2 forward: 5′-GCGAGATCTACCATGGCTCCCAGCGTGCCAGC,

AK2 reverse: 5′-GCGCTCGAGATGATAAACATAACCAAGTCTTTACATGTGG,

fNEAP cDNA forward: 5′-GCGGGATCCACCATGGCGTTTATGTCCAGATTGTCTC

fNEAP cDNA reverse: 5′-GCGCTCGAGATTGTGGTGCTGCGGCTGCTG

fDUSP23 forward: 5′-GTGCGCCCACATTTGAGCAGATC,

fDUSP23 reverse: 5′-CTGCACAATCATTTGTTCCTGCTCTC,

zebrafish 18S rRNA forward: 5′-CCGCAGCTAGGAATAATGGA,

zebrafish 18S rRNA reverse: 5′-CATCGTTTACGGTCGGAACT,

fNEAP morpholino sequence is 5′-GAGACAATCTGGACATAAACGCCAT,

fNEAP scrambled morpholino is 5′-CCATAATGGACATGAGATCACAACG.

### Data availability statement

All data generated or analyzed during this study are included in this published article.

## Electronic supplementary material

Supplementary Information
