# Supplementary material for: NEAP/DUSP26 suppresses receptor tyrosine kinases and regulates neuronal development in zebrafish
Source: Sci Rep. 2017 Jul 12;7:5241. doi: 10.1038/s41598-017-05584-7 (PMC5507855; doi:10.1038/s41598-017-05584-7)

Supplementary Information

NEAP/DUSP26 suppresses receptor tyrosine kinases and regulates neuronal development in zebrafish

Chi-Hwa Yang[1,4], Yu-Jung Yeh[1,4], Jiz-Yuh Wang[1,3,4], Ya-Wen Liu[1], Yen-Lin Chen[1], Hui-Wen Cheng[1], Chun-Mei Cheng[1], Yung-Jen Chuang[2], Chiou-Hwa Yuh[1], Yi-Rong Chen[1,5]

[1]Institute of Molecular and Genomic Medicine, National Health Research Institutes, Zhunan, Taiwan 350
[2]Institute of Bioinformatics and Structural Biology, National Tsing Hua University, Hsinchu, Taiwan 300
[3]Present affiliation: Graduate Institute of Medicine, College of Medicine, Kaohsiung Medical University, Taiwan 807

[4]The first three authors contributed equally to this work.

[5]Correspondence and request for materials should be addressed to YR Chen (yrchen@nhri.org.tw)

Supplementary materials for Figure 1a

Expt. 2

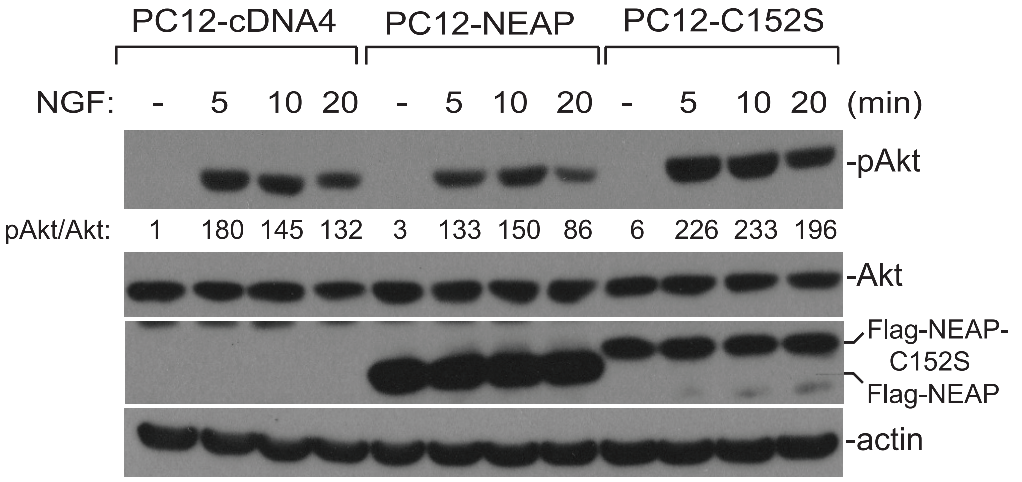

Expt. 3

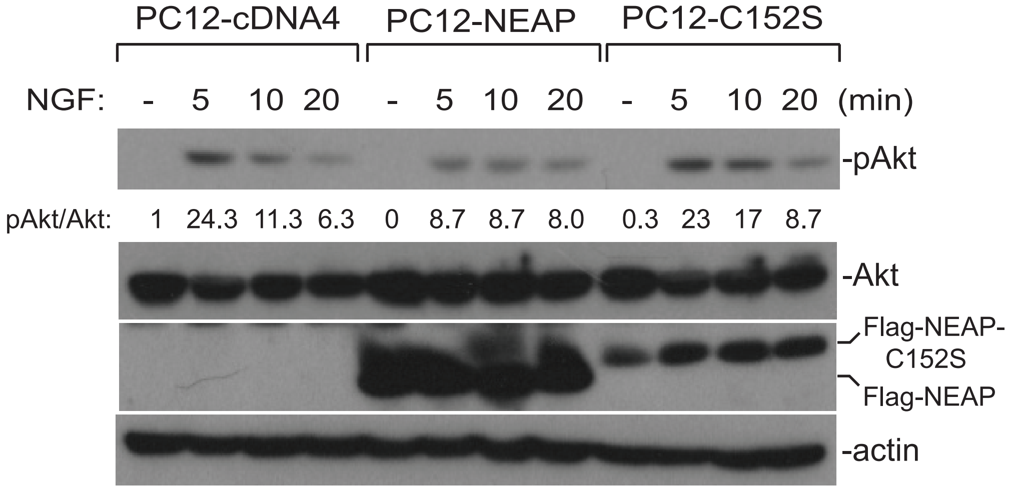

Supplementary materials for Figure 1b

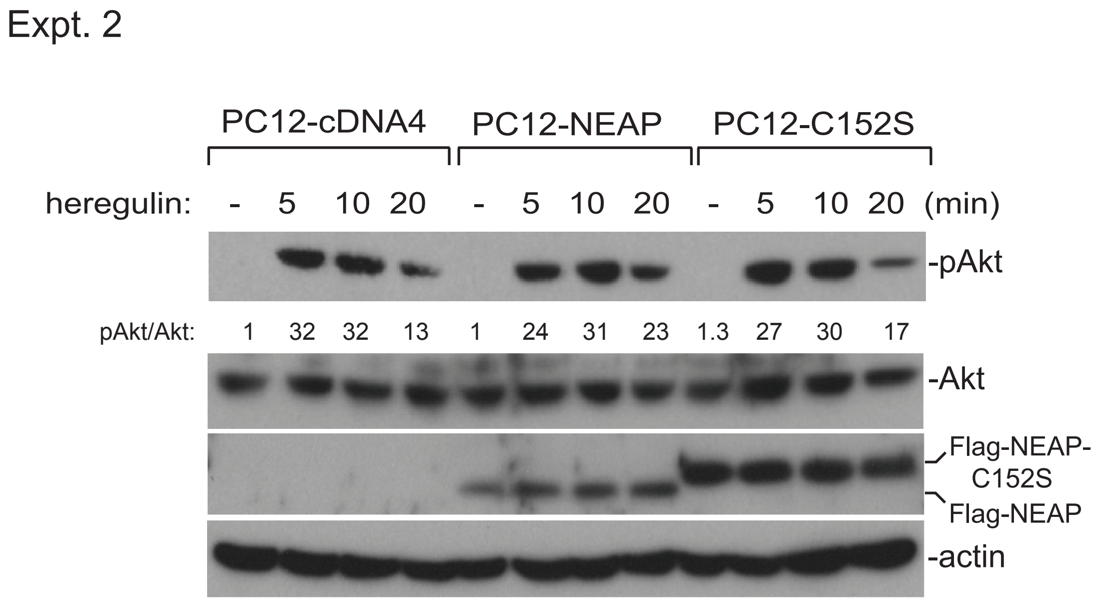

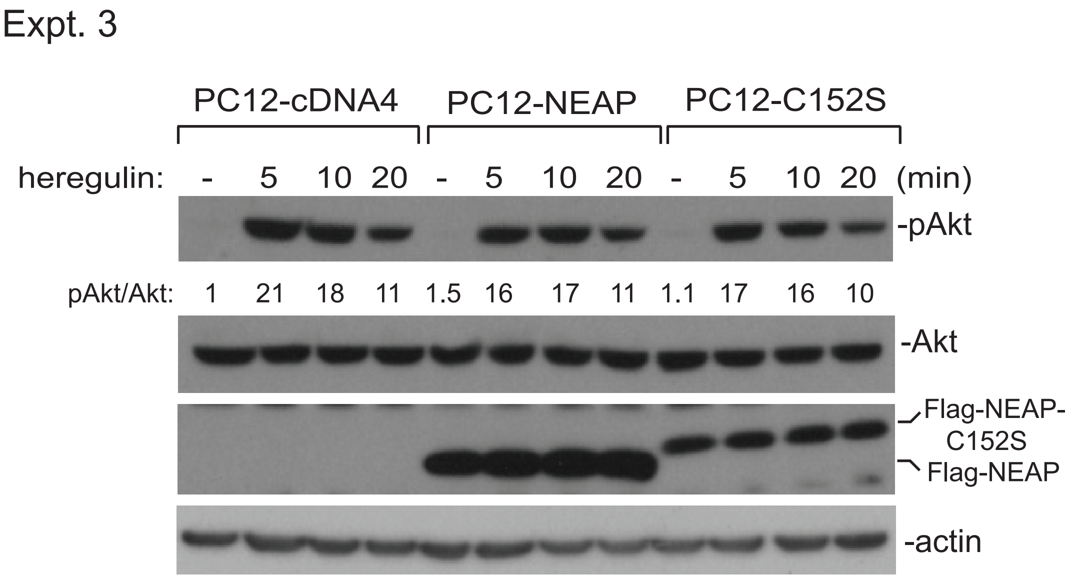

Supplementary materials for Figure 2

Expt. 2

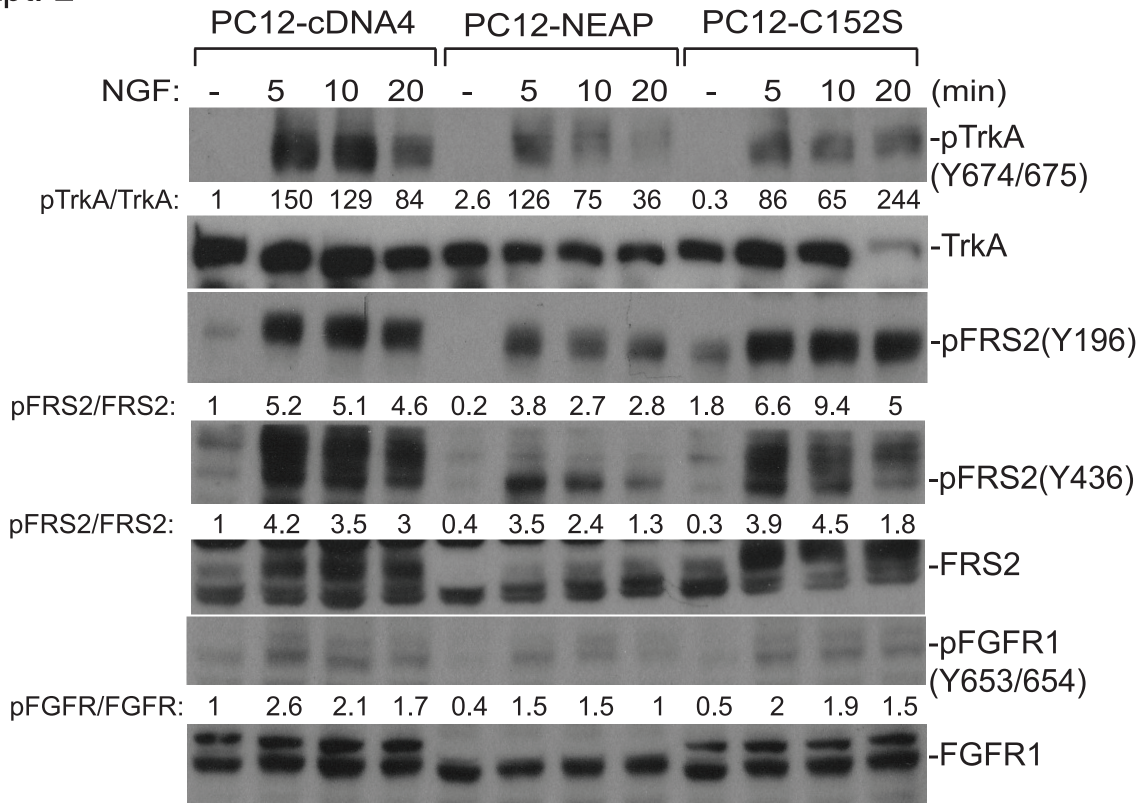

Expt. 3

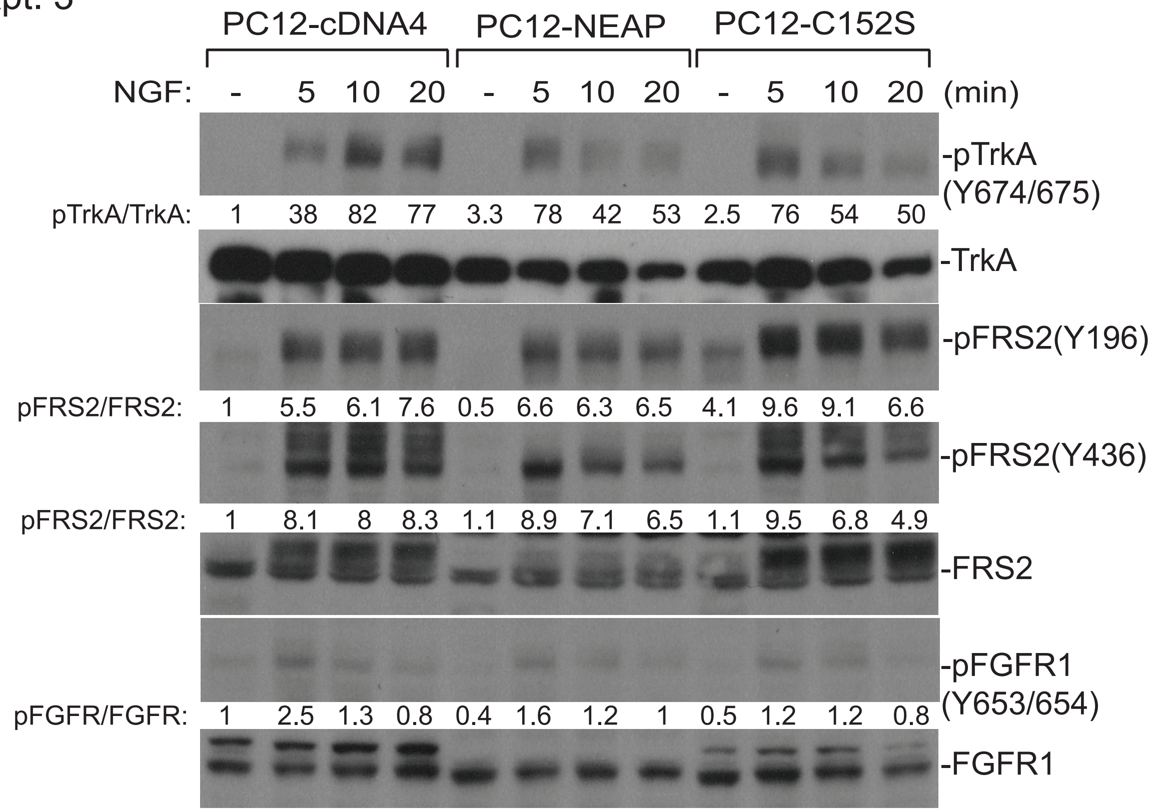

Supplementary materials for Figure 3a

Expt. 2

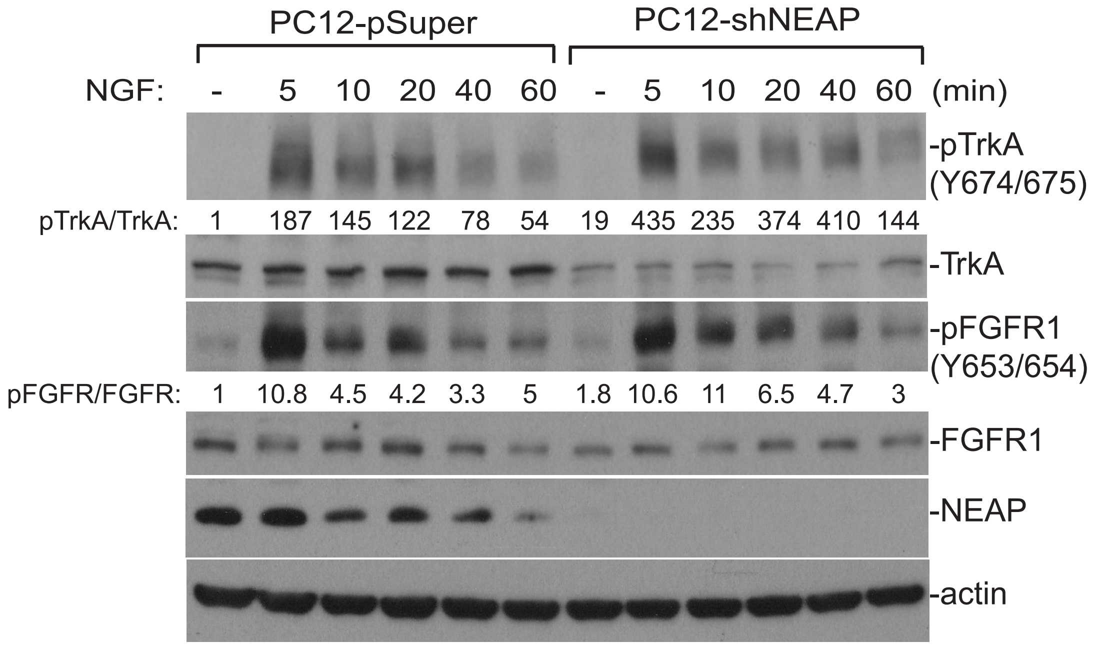

Expt. 3

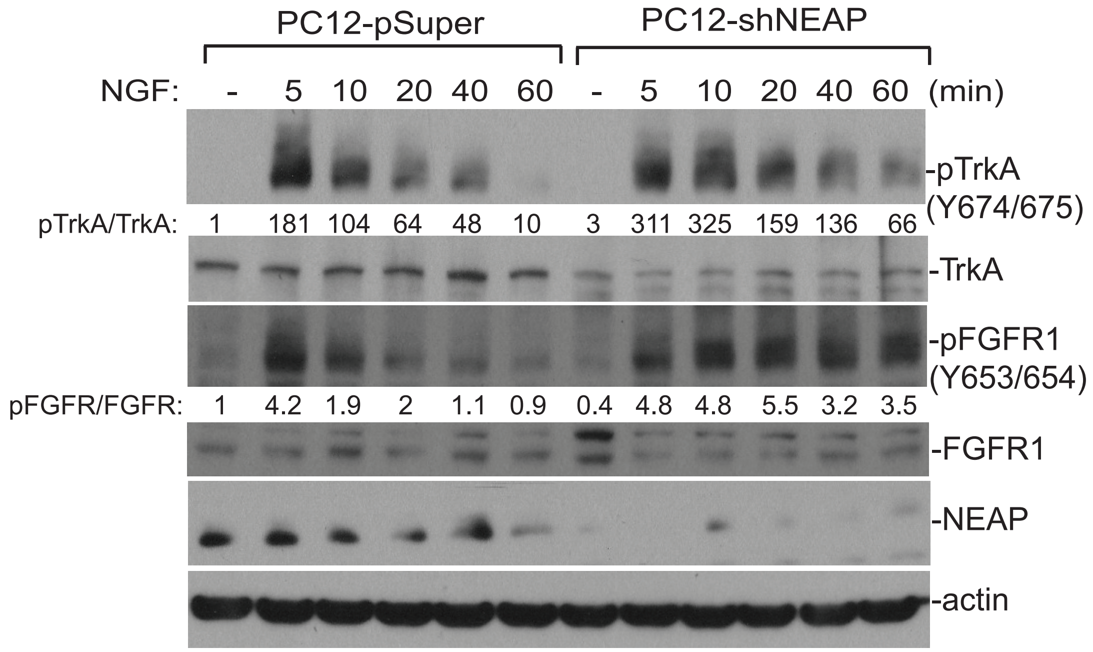

Supplement: Supplementary file 1 — Supplementary Information [file 41598_2017_5584_MOESM1_ESM.pdf]
